# Systematic review and meta-analysis of percutaneous nephrolithotomy in flank versus prone position

**DOI:** 10.1186/s12894-024-01544-2

**Published:** 2024-07-29

**Authors:** Qing He, Liqiang Xiong, Renbo Wei, Lei Fu, Liang Zhou, Renbin Yuan, Hui Zhuo

**Affiliations:** Department of Urology, The Third People’s Hospital of Chengdu, Southwest Jiaotong University, No. 82 Qinglong Street, Chengdu, Sichuan 610031 P.R. China

**Keywords:** Percutaneous nephrolithotomy, Flank position, Prone position, Systematic review and meta-analysis

## Abstract

**Background:**

This systematic review and meta-analysis aimed to evaluate the efficiency and safety of percutaneous nephrolithotomy (PCNL) between flank position and prone position for the treatment of renal stones.

**Methods:**

PubMed, Embase, OVID, and Cochrane Library were comprehensively searched from their inception to Jul 2024. Randomized and nonrandomized trials evaluating renal calculi patients who underwent PCNL via flank position or prone position were included. Data extraction and quality assessment were conducted by two independent reviewers. The outcomes and complications of both groups were compared in this meta-analysis.

**Results:**

This review involved five articles (554 patients). Specifically, four articles were randomized controlled trials, and the remaining publication was prospective cohort study. No significant difference was found in stone-free rate between the flank group and prone group after the PCNL procedure. Similarly, the percutaneous access time, operative time, and hospital stay of flank position had no significant difference compared with the prone group. There was no significant difference in the comparison of complication rates between the flank group and the prone group. Although further analysis indicated that patients in the prone position suffered more hemoglobin drop than the flank group, no significant difference was found in the hemorrhage and blood transfusion rates.

**Conclusions:**

Both surgical positions were appropriate for most PCNL procedures and had shown similar efficacy and safety. In practice, the optimal choice should be made according to the patients’ conditions and urologists’ acquaintance.

## Background

Percutaneous nephrolithiasis (PCNL), as one of the minimal invasive lithotripsies, is recommended to manage renal stones more than 20 mm and staghorn calculi. Since Fernstrom and Johansson successfully extracted renal stone via nephrostomy tract in 1976 [1], PCNL is mostly conducted in the prone position. The prone position provides a large surface area for renal track formation and allows urologists to manipulate the nephroscope in a wide space. It also reduces the risk of abdominal visceral injuries and facilitates the puncture of the upper pole of the kidney [2]. In practice, however, the prone position does have some disadvantages. First, it may increase the operative time during patient repositioning and may compromise the patient’s airway access. Lying on the abdomen leads to abdominal compression and reduces lung compliance and cardiac output [3]. It is difficult for anesthesiologists to handle eventual cardiorespiratory emergencies.

The flank position is practical for PCNL, especially in obese, kyphotic, and high-risk patients, as urologists are familiar with this position applied in open and laparoscopic renal surgery. The operative table is curved, which widens the space between the 12th rib and the iliac crest, flattens the folds of adipose tissue, and facilitates percutaneous puncture. Furthermore, since the patient is not prone, the procedure can be conducted safely even under regional anesthesia, avoiding the risks of general anesthesia [4]. It is a remarkable fact that the fluoroscopic view of the kidney is unusual and may also be obscured by the spine below in this position [5]. Similarly, patients also experience repositioning, but it is easier and has a lower risk than the prone position.

Both positions seem to pose their own advantages and disadvantages. Although some studies have reported the comparison results between flank position and prone position in PCNL for treatment of renal stones, the conclusions were not consistent. Thus, to evaluate the efficacy and safety of PCNL in flank versus prone position for the management of renal calculi, we carried out this systematic review and meta-analysis.

## Methods

### Search strategy

According to the Preferred Reporting Items for Systematic Reviews and Meta-Analyses (PRISMA) checklist, we retrieved literature from databases including PubMed, Embase, OVID and Cochrane library (up to Jul 2024). No limitation in the language of publication was applied. We used the Boolean operator “and” to combine the search themes. First, the theme *PCNL* and expanded versions of Medical Subject Headings (MeSH) terms *percutaneous nephrolithotomy* and *percutaneous nephrolithotomies* were combined with the Boolean operator “or”. Second, the theme *prone* with the expanded versions of MeSH terms *prone position* or *prone positions* were retrieved. The last theme was the *flank position*, combining the synonyms *flank positions*, *lateral position*, or *lateral positions*. We also screened the reference lists of all selected publications to identify additional articles.

### Inclusion and exclusion criteria

Two reviewers independently reviewed the potentially relevant articles according to the following inclusion criteria: (1) randomized controlled trials (RCTs) or comparative studies evaluating the efficacy and safety of the flank position versus prone position in the treatment of adult renal stones by PCNL; and (2) studies reporting at least one outcome of interest, for example, stone-free rate (SFR), complication, access time, operative time, hospital stay, and hemoglobin change. In addition, studies were excluded if (1) published as abstracts, comments, reviews, case reports, and studies were unpublised; and (2) publications including patients who were pregnant women or children, had complete staghorn stones, had other urinary anomalies (horseshoe kidney, duplex kidney, ectopic kidney, etc.), had active urinary tract infection, or received renal surgery previously. We only selected the latest data from duplicated studies that enrolled the same population.

### Data extraction and quality assessment

Two reviewers independently extracted variables including the first author’s name, year of publication, study type and period, sample size, age, gender, body mass index, stone size and location, assessment methods and criteria, follow-up time, SFR, adverse events, percutaneous access time, operative time, and hospital stay. And the detailed complications were classified according to the Clavien-Dindo classification of surgical complications. Dichotomous data were collected using two-by-two tables. For continuous data, available summary estimates for both groups (means, changes in means) and measures of variability (standard deviation, 95% confidence interval [CI]) were extracted. The levels of evidence (LE) of all included studies were assessed according to the Oxford Centre for Evidence-Based Medicine-Levels of Evidence [6]. The methodological quality of the studies was evaluated according to the Modified Jadad Scale [7] for RCTs and the Newcastle-Ottawa Scale [8] for nonrandomized controlled trials.

### Statistical analysis

All of the statistical analyses were conducted by using RevMan 5.4 software (Cochrane Collaboration, Oxford, UK). The treatment outcomes of PCNL were assessed by risk ratio (RR) and mean difference (MD) with a corresponding 95% CI under the comparison between the flank position group and prone position group. And if *P* < 0.05, the difference was considered statistically significant. The Cochrane Q statistic (significance level of *P* ≤ 0.10) and the inconsistency (*I*^2^) test were used to assess the heterogeneity among studies. If the heterogeneity was significant, we used the random-effects model to generate the most conservative estimate. Otherwise, the fixed-effects model was used to pool the data. The counting data was statistically analyzed using the Mantel-Haenszel method, and the quantitative data was analyzed using the Inverse Variance method. Sensitivity analysis was performed to evaluate the stability of the pooled data.

## Results

### Study identification and characteristics

**Fig. 1:**
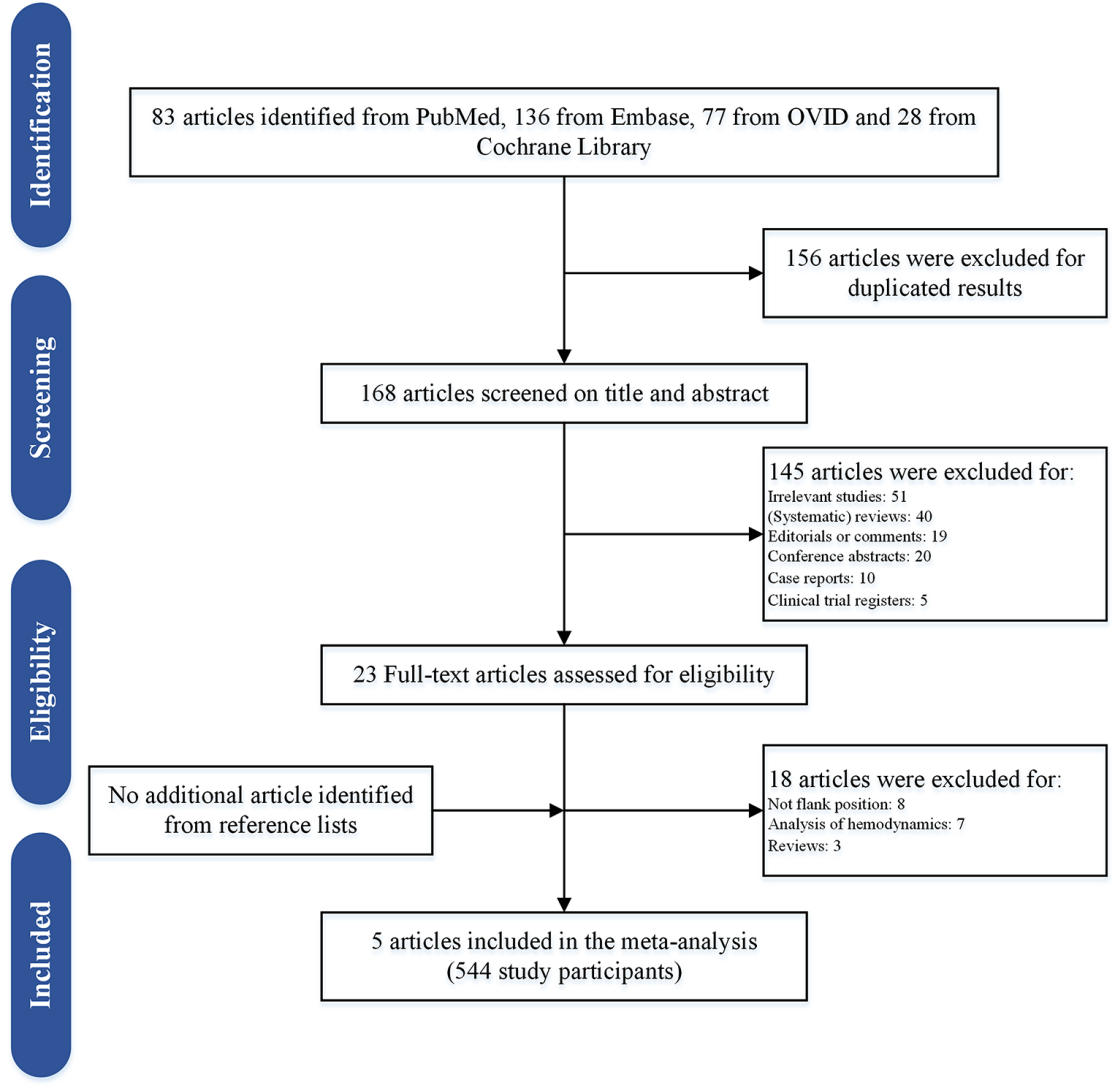
PRISMA flow diagram of study selection

As summarized in Fig. 1, only five publications [9–13] were included in this systematic review after screening abstracts, full-text articles, and reference lists, and no additional studies were identified by scanning the references lists in these articles. Among them, four articles [10–13] were RCTs (LE: 1b), and one publication [9] was prospective cohort study (LE: 2b). We extracted unduplicated and useful data from these articles.

A total of 544 patients were enrolled in this meta-analysis with a ratio of 1:1 between the flank group and prone group. All the patients in flank group were positioned in a standard flank position except for one study [13] where PCNL was performed in a split-leg modified lateral position. All included studies indicated that all PCNL procedures were performed under general anesthesia. In the flank group, three studies [9–11] determined that the puncture of the caliceal system was guided by ultrasound. On the contrary, the renal access of the prone group was mostly established under fluoroscopic guidance. The follow-up duration ranged from two weeks to three months. Only one study [9] used the tubeless PCNL procedure, but all included studies used the pneumatic lithotripsy for stone fragmentation. The baseline characteristics of the participants are summarized in Table 1.

**Table 1:** Characteristics of studies included in the meta-analysis

Study	Level of evidence	Study design	Study quality	Study period	Group	Sample size	Age, mean (SD)	Gender		BMI, mean (SD), kg/m^2^	Side		Stone size, mean (SD), mm	Stone location					Number of stones, mean (SD)	Hydronephrosis			Detecting tools of puncture	Assessment tools of residual fragments	Definition of stone free	Follow-up period	Percutaneous access			Number of punctures		Postoperative drainage
								Male (%)	Female (%)		Right (%)	Left (%)		Superior calix (%)	Middle calix (%)	Inferior calix (%)	Renal pelvis (%)	Multiple (%)		Normal or mild (%)	Moderate (%)	Severe (%)					Superior calyx (%)	Middle calyx (%)	Inferior calyx (%)	1	2	
Karami, 2010	2b	Prospective cohort study	5^a^	2007-2008	Flank	30	40.8 (6.9)	18 (60)	12 (40)	27.8 (3.4)	22 (73.3)	8 (26.7)	28.7 (3.3)	3 (10)	7 (23.3)	14 (46.7)	6 (20)	0	2 (1.1)	12 (40)	16 (53.3)	2 (6.7)	Ultrasound	Ultrasound, KUB	No stone ≥ 4 mm	1 month	1 (3.3)	5 (16.7)	24 (80)	30	0	Tubeless
					Prone	30	39.4 (10.6)	19 (63.3)	11 (36.7)	26.7 (4.7)	16 (53.3)	14 (46.7)	27.4 (3.2)	2 (6.7)	7 (23.3)	17 (56.7)	4 (13.3)	0	2.1 (1.5)	14 (46.7)	11 (36.7)	5 (16.7)	Fluoroscopy				1 (3.3)	3 (10)	26 (86.7)	30	0	
Karami, 2013	1b	RCT	3^b^	2010-2011	Flank	50	40.7 (8.4)	31 (62)	19 (38)	27 (4.5)	24 (48)	26 (52)	27.5 (3.6)	1 (2)	1 (2)	5 (10)	13 (26)	30 (60)	2.1 (1)	18 (36)	25 (50)	7 (14)	Ultrasound	CT	No stone > 3 mm	1 month	1 (2)	4 (8)	45 (90)	50	0	Tubeless
					Prone	50	41.5 (8.8)	31 (62)	19 (38)	26.1 (4.1)	26 (52)	24 (48)	28.3 (3.6)	1 (2)	2 (4)	7 (14)	12 (24)	28 (56)	2.3 (1.2)	22 (44)	24 (48)	4 (8)	Fluoroscopy				1 (2)	6 (12)	43 (86)	50	0	
Radfar, 2021	1b	RCT	7^b^	2017-2019	Flank	100	42.3 (6.6)	61 (61)	39 (39)	26.6 (6.3)	52 (52)	48 (48)	27.1 (3)	4 (4)*	18 (18)*	31 (31)*	47 (47)*	47 (47)	2.3 (1.4)	45 (45)	50 (50)	5 (5)	Ultrasound	Ultrasound, KUB, CT	No stone ≥ 4 mm	3 months	NA			100	0	Nephrostomy tube
					Prone	100	44 (7.2)	54 (54)	46 (46)	25.1 (5.2)	56 (56)	44 (44)	27.8 (3.4)	9 (9)*	23 (23)*	21 (21)*	47 (47)*	52 (52)	2.1 (1.2)	51 (51)	43 (43)	6 (6)	Ultrasound							100	0	
Hosseini, 2021	1b	RCT	4^b^	2020-2021	Flank	31	47.5 (7.2)	16 (51.6)	15 (48.4)	31.4 (1.1)	NA		32 (6.9)	4 (12.9)	8 (25.8)	8 (25.8)	11 (35.5)	0	1.7 (0.8)	NA			Fluoroscopy	CT	No stone ≥ 4 mm	3 months	NA			31	0	Ureter catheter
					Prone	29	47.7 (9.2)	18 (62.1)	11 (37.9)	31 (0.9)			31.3 (9.7)	2 (6.9)	4 (13.8)	6 (20.7)	17 (58.6)	0	1.5 (0.7)				Fluoroscopy							29	0	
Ahmed, 2021	1b	RCT	6^b^	2017-2019	Flank	61	46.5 (13.5)	38 (62.3)	23 (37.7)	28.6 (4.02)	25 (41)	36 (59)	28.3 (10)	23			6	32	NA	43 (70.5)	14 (23)	4 (6.6)	Fluoroscopy	CT	No stone > 3 mm	2 weeks	9 (13.6)	20 (30.3)	37 (56.1)	56 (91.8)	5 (8.2)	Nephrostomy tube
					Prone	63	45.6 (9.5)	45 (71.4)	18 (28.6)	27.1 (2.8)	33 (52.4)	30 (47.6)	32.9 (4.6)	31			10	22		35 (55.6)	22 (34.9)	6 (9.5)	Fluoroscopy				14 (19.7)	24 (33.8)	33 (46.5)	55 (87.3)	8 (12.7)	

*SD* standard deviation, *BMI* body mass index, *KUB* kidney, ureter, and bladder, *CT* computed tomography, *RCT* randomized controlled trial, *NA* not available

Study quality assessed by ^a^the Newcastle-Ottawa Scale and ^b^the modified Jadad score

*Stone location is defined as the calyx (or pelvis) that more than 50% bulk of the stone is located there

### Meta-analysis outcomes

#### Stone-free rate and auxiliary procedure rate

**Fig. 2:**
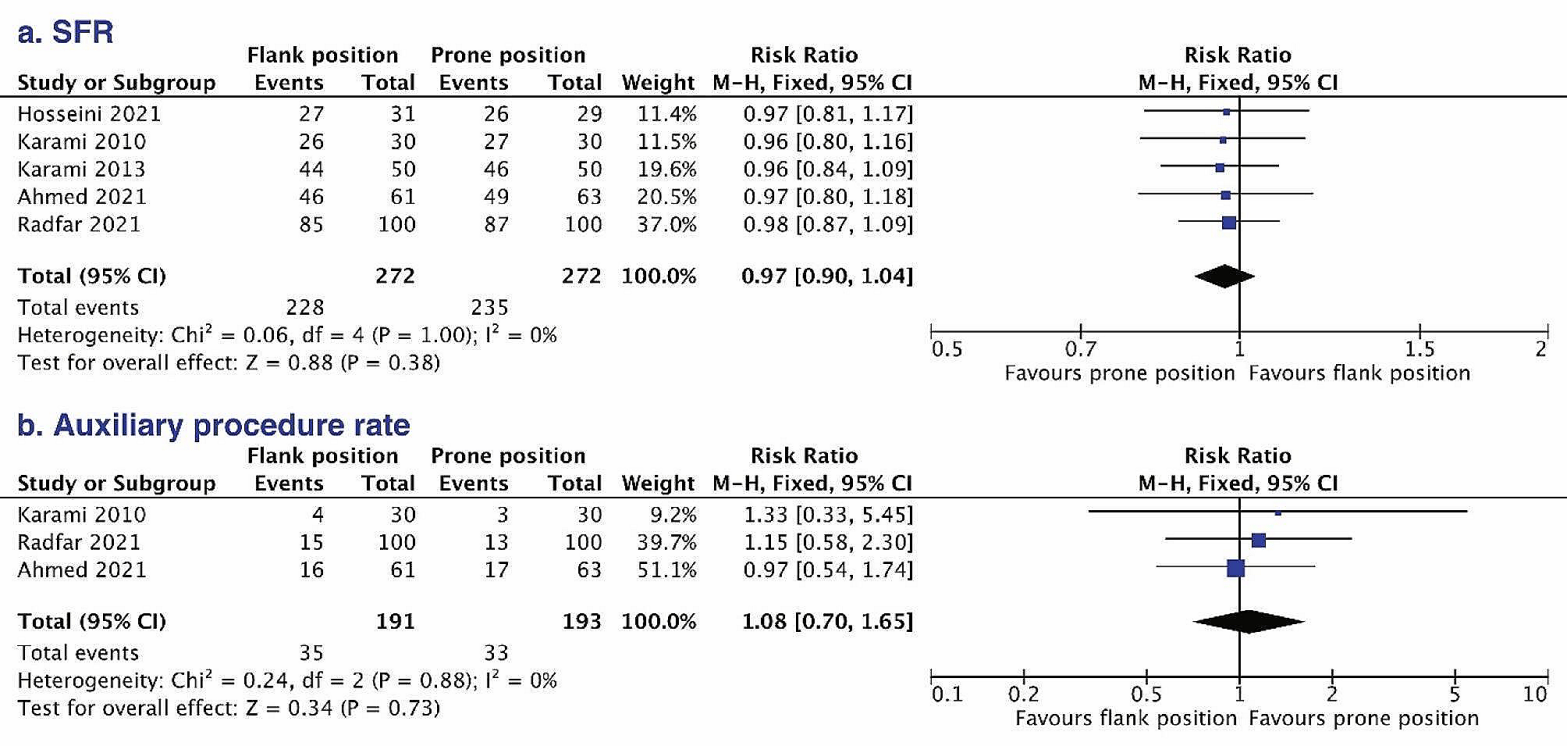
Forest plots comparing stone-free rate (**a**) and auxiliary procedure rate (**b**) between the flank position and prone position

All included studies enrolling 544 patients reported SFR, but the definition of stone-free was different among them. Specifically, three publications defined stone free as residual fragments less than 4 mm, and the rest of them defined stone free as no stone larger than 3 mm. In total (Fig. 2a), the SFR was comparable (RR = 0.97, 95% CI 0.90 to 1.04) between flank group (83.8%) and prone group (86.4%). The results of the Cochrane Q statistic (*P* < 0.01) and *I*^2^ test (0%) could exclude significant heterogeneity. Similarly, the auxiliary procedure rate of flank position (18.3%) had no significant difference (RR = 1.08, 95% CI 0.70 to 1.65) compared with the prone group (17.1%) (Fig. 2b). The detailed auxiliary procedures are summarized in Table 2.

**Table 2:** Summary of detailed auxiliary procedures of included studies

Study	Group	ESWL, *n*(%)	PCNL, *n*(%)	RIRS, *n*(%)
Karami, 2010	Flank	4	0	0
	Prone	3	0	0
Radfar, 2021	Flank	12	3	0
	Prone	8	5	0
Ahmed, 2021	Flank	13	2^*^	1
	Prone	12	1	4

ESWL extracorporeal shock wave lithotripsy, PCNL percutaneous nephrolithiasis, RIRS retrograde intrarenal surgery

^*^Combined PCNL and RIRS

#### Access time, operative time and hospital stay

**Fig. 3:**
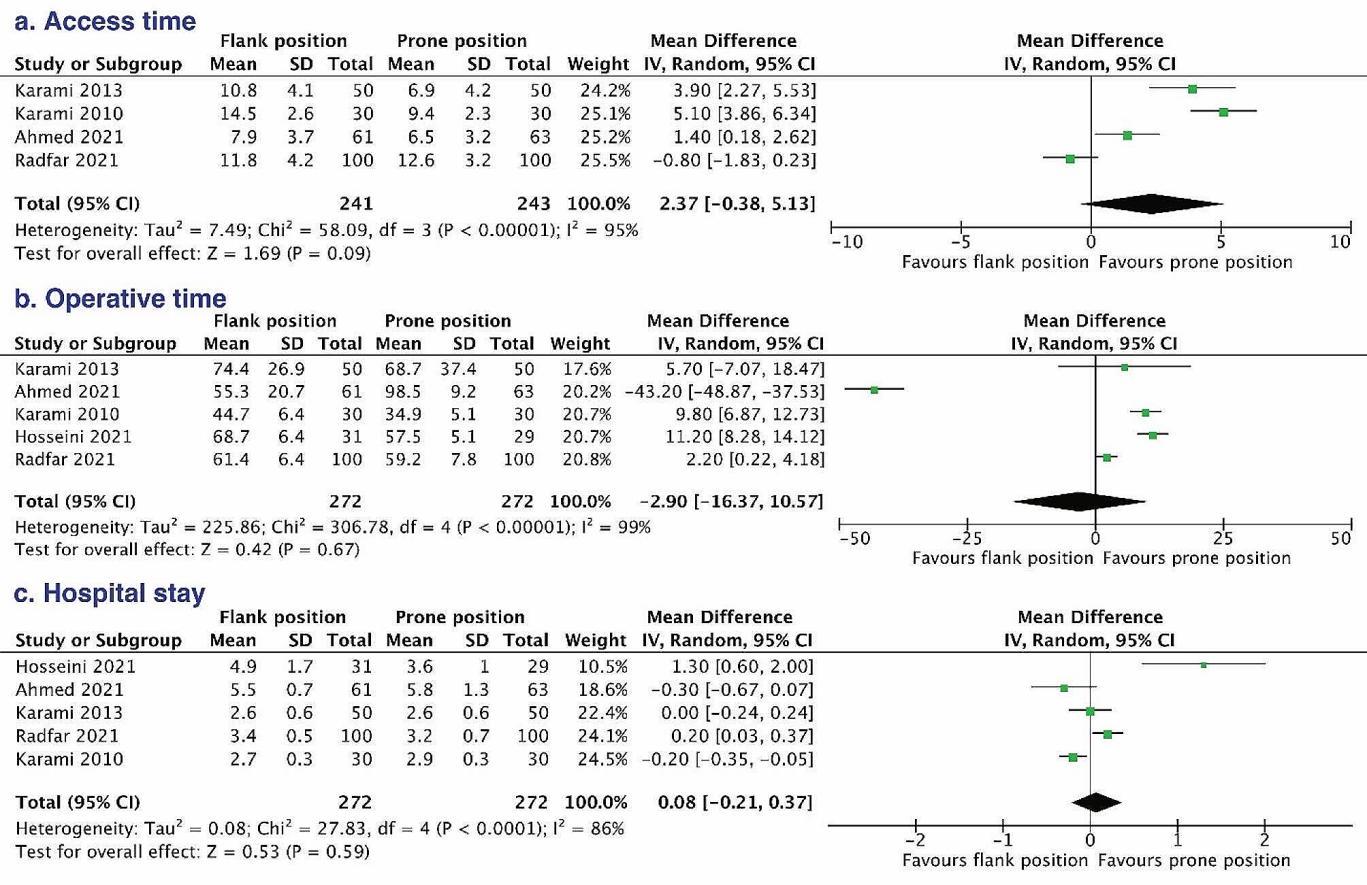
Forest plots of the comparisons of access time (**a**), operative time (**b**), and hospital stay (**c**) between the flank position and prone position

All three pooled outcomes were generated via the random-effects model due to significant heterogeneity (Fig. 3). Intriguingly, no significant difference in the comparison of percutaneous access time (MD = 2.37, 95% CI – 0.38 to 5.13), operative time (MD = – 2.90, 95% CI – 16.37 to 10.57), and hospital stay (MD = 0.08, 95% CI – 0.21 to 0.37) was found between the flank group and prone group, although some studies [9, 13] indicated that flank group would significantly consume more time in track formation and operation. Due to the large heterogeneity among the studies, sensitivity analyses that excluded a single study and calculated the pooled MD for remaining studies had unstable results of percutaneous access time and operative time (Table 3). Patients in flank position spent more time on the establishment of the percutaneous renal tract (MD = 3.45, 95% CI 1.12 to 5.79) and the length of the operation (MD = 7.42, 95% CI 1.90 to 12.94) when the single study was omitted. However, the sensitivity analysis of hospital stay had similar converged value of MD and 95% CI by omitting each study.

#### Complications

**Fig. 4:**
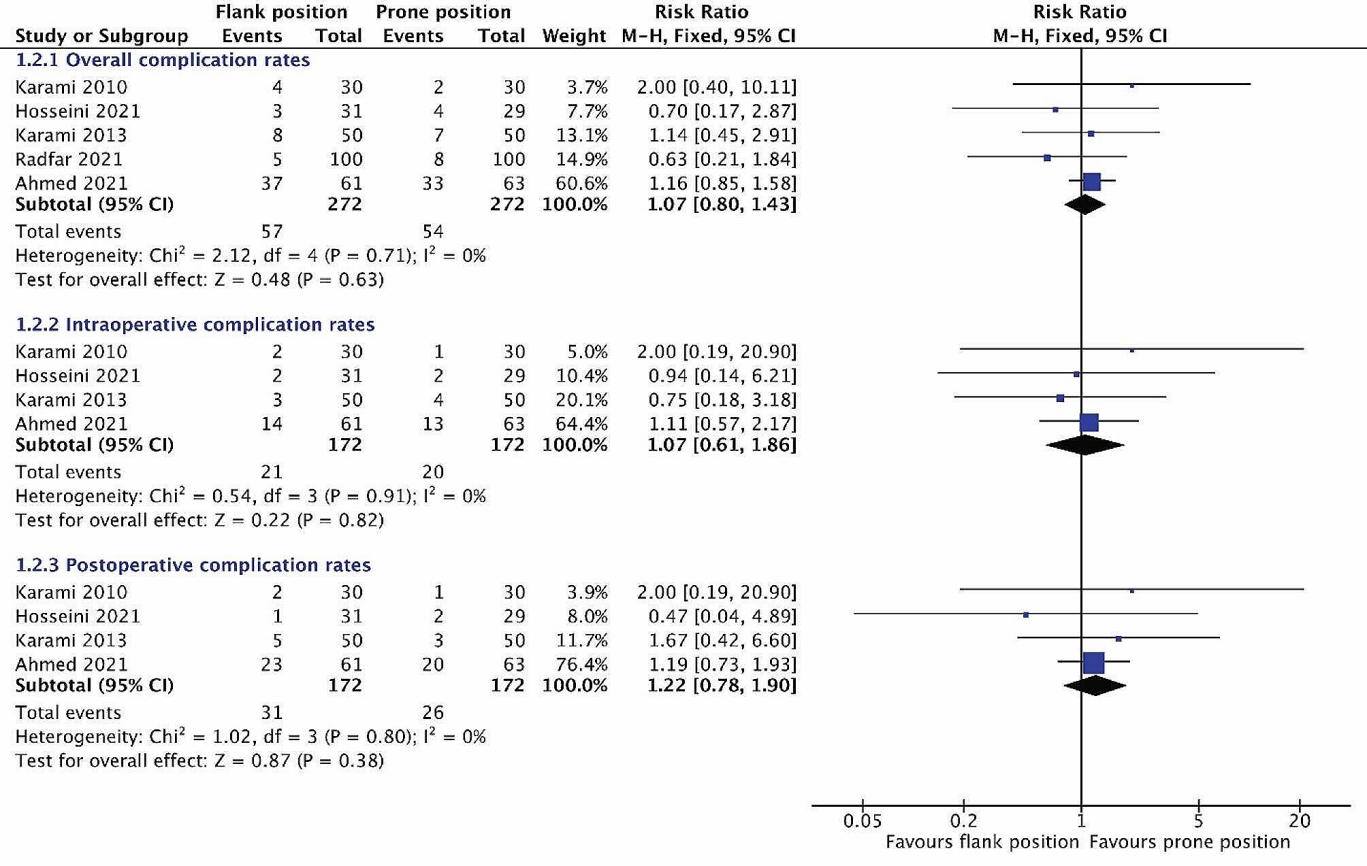
Forest plots comparing overall complication rates (**a**), intraoperative complication rates (**b**), and postoperative complication rates (**c**) between the flank position and prone position

There was no significant difference in the overall complication rates between the two groups (RR = 1.07, 95% CI 0.80 to 1.43) (Fig. 4). Furthermore, we divided complications into two parts, intraoperative complications and postoperative complications. And further analyzed results showed, similar to the overall complication rates, the intraoperative complication rates (RR = 1.07, 95% CI 0.61 to 1.86) and postoperative complication rates (RR = 1.22, 95% CI 0.78 to 1.90) were not significantly different between two positions.

**Fig. 5:**
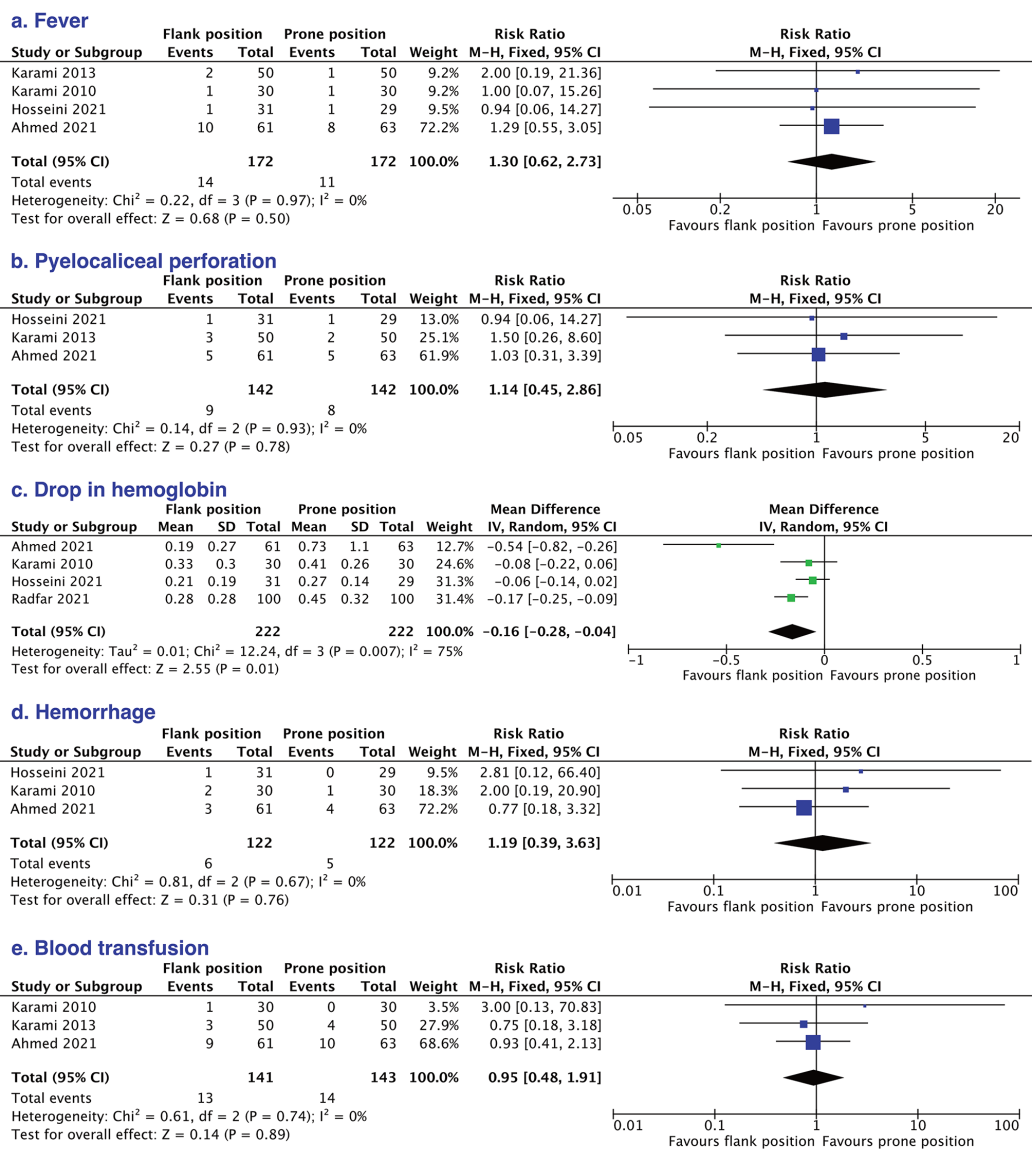
Forest plots of the comparisons of the pooled incidence of fever (**a**), pyelocaliceal perforation (**b**), drop in hemoglobin (**c**), hemorrhage (**d**), and blood transfusion (**e**) between the flank position and prone position

Finally, we pooled the data of each complication, which indicated no significant difference between the two groups in the rate of fever (RR = 1.30, 95% CI 0.62 to 2.73) (Fig. 5a) and the rate of pyelocaliceal perforation (RR = 1.14, 95% CI 0.45 to 2.86) (Fig. 5b). Although flank position presented a lower risk in hemoglobin reduction (MD = – 0.16, 95% CI – 0.28 to – 0.04) with a large heterogeneity (*P* = 0.007, *I*^*2*^ = 75%) (Fig. 5c), insignificant difference was found in the rates of hemorrhage (RR = 1.19, 95% CI 0.39 to 3.63) (Fig. 5d) and blood transfusion (RR = 0.95, 95% CI 0.48 to 1.91) (Fig. 5e). The sensitivity analysis of hemoglobin reduction showed similar pooled results after omitting each study (Table 3). The detailed complications extracted from the included studies are listed in Table 4.

**Table 3:** Summary of sensitivity analyses

Results	Omitted Study	MD [ 95% CI]
Access time		2.37 [-0.38, 5.13]
	Karami, 2010	1.44 [-1.11, 3.98]
	Karami, 2013	1.89 [-1.51, 5.28]
	Ahmed, 2021	2.71 [-1.24, 6.66]
	**Radfar**,** 2021**	**3.45 [1.12**,** 5.79]**
Operative time		-2.90 [-16.37, 10.57]
	Karami, 2010	-6.17 [-24.52, 12.19]
	Karami, 2013	-4.75 [-19.78, 10.29]
	**Ahmed**,** 2021**	**7.42 [1.90**,** 12.94]**
	Hosseini, 2021	-6.53 [-24.53, 11.46]
	Radfar, 2021	-4.19 [-25.77, 17.38]
Hospital stay		0.08 [-0.21, 0.37]
	Karami, 2010	0.18 [-0.18, 0.55]
	Karami, 2013	0.13 [-0.26, 0.52]
	Ahmed, 2021	0.17 [-0.16, 0.50]
	Hosseini, 2021	-0.06 [-0.28, 0.17]
	Radfar, 2021	0.06 [-0.31, 0.42]
Drop in hemoglobin		-0.16 [-0.28, -0.04]
	Karami, 2010	-0.20 [-0.36, -0.03]
	Ahmed, 2021	-0.11 [-0.18, -0.03]
	Hosseini, 2021	-0.22 [-0.39, -0.04]
	Radfar, 2021	-0.18 [-0.38, -0.02]

MD mean difference, CI confidence interval

**Table 4:** Summary of detailed complications of included studies

Study	Group	Complications, n								Clavien-Dindo grade, n		
		Total	Fever	Urinary leakage	Hematoma or Hematuria	Hemorrhage	Blood transfusion	Injury to adjacent organs	Pyelocaliceal perforation	I	II	III
Karami, 2010	Flank	4	1	0	0	2	1	0	0	2	2	0
	Prone	2	1	0	0	1	0	0	0	1	1	0
Karami, 2013	Flank	8	2	0	0	0	3	0	3	0	5	3
	Prone	7	1	0	0	0	4	0	2	0	5	2
Radfar, 2021	Flank	5	NA							4	1	0
	Prone	8								5	2	1
Hosseini, 2021	Flank	3	1	0	0	1	0	0	1	1	1	1
	Prone	4	1	0	1	0	0	1	1	2	0	2
Ahmed 2021	Flank	37	10	10	0	3	9	0	5	13	19	5
	Prone	33	8	8	0	4	10	0	3	12	18	3

## Discussion

Traditionally, PCNL is mostly performed in the prone position. Due to the reasons of anesthetic concerns, urologists persist in exploring alternative surgical positions. The flank position, as a familiar position for urologists, may not only reduce the hemodynamic and respiratory risks but also increase the patient’s comfort and safety [14]. The meta-analysis results illustrated that the flank position group presented a similar SFR, auxiliary procedure rate, percutaneous access time, operative time, hospital stay, and complication rate compared with the prone group. The fewer hemoglobin drops indicated that PCNL could be safely and effectively performed in the flank position.

Our study indicated that SFR was comparable between the two positions, although the working tract is nearly vertical to the operating table in the flank position, which may limit the evacuation of stone fragments. In our opinion, this comparable result might be attributed to the following reasons. Firstly, the space between the 12th rib and the iliac crest can be increased with the flex of the operating table after the patient is placed in the lateral position, which also provides a wider manipulate space like the prone position. Secondly, the modified lateral position offers the possibility of simultaneous ureteroscopic and nephroscopic procedures [13], which could contribute to the increase of SFR. Furthermore, due to the effects of gravity, vibration, and water flow, the stones in the renal calyx are more likely to fall into the renal pelvis in the lateral position, which makes it easier to clear residual stones.

In our review, the pooled data indicated that the outcomes of percutaneous time, operative time and hospital stay were not significantly different between the prone and flank position groups with a large heterogeneity. Our sensitivity analyses revealed unstable results of percutaneous access time and operative time (Table 3). Indeed, some studies [9, 13] indicated that flank group would significantly consume more time in track formation and operation. Generally, the lateral position is inappropriate for percutaneous guided by a C-arm due to the obscurity of the spine below. Indeed, the percutaneous tract of the flank group was mostly established under ultrasonic guidance [5]. However, the split-leg modified lateral position enables C-arm-guided renal track formation [13]. The lateral position is a traditional open surgery position. The urologists are more acquainted anatomy of the kidney and adjacent organs, and it is easier to grasp the puncture angle and depth. On the other hand, with the kidney is being more accessible when the patient is placed in the flank position, the renal percutaneous tract can be established easily under ultrasonic guidance with better vision [15]. In other words, ultrasound-guided PCNL could avoid ionizing radiation, hence it is a safe efficient modality, especially for pediatric patients [16]. Since the standard prone position and flank position were both transformed from the lithotomy position, the operative time was comparable between the two groups. However, the modified lateral position could significantly save operative time by avoiding the repositioning, re-prepping, and re-draping of patients as well as the re-scrubbing and gowning of urologists [13]. Another advantage of this position is that simultaneous ante- and retrograde renal or ureteral access allows the extraction of complex upper urinary tract stones [17]. Nonetheless, this position is not suited for every patient since it requires musculoskeletal mobility and flexibility of the spine [18]. Using flexible instruments, simultaneous percutaneous and transurethral access has also been achieved in the modified prone position [19, 20], although this can be challenging to most urologists. Consistent with our meta-analysis, most of the previous studies reported similar lengths of hospital stay in different patients’ positioning groups [21, 22].

Complication rates, as the key elements of the safety assessment, are related to the cost and prognosis of patients. In our pooled data, the rates of overall complication, intraoperative complication, and postoperative complication were all insignificantly different between the two groups. Although the hemoglobin drop was significantly higher in the prone position, the comparison outcomes of hemorrhage and blood transfusion were similar between the two groups. It might be associated with the shorter operative time of the modified lateral position group accompanied by less blood loss [13]. This might be the cause, as there was no significant difference in the amount of hemoglobin decline between the two groups found in the other included studies. The establishment of the percutaneous renal tract is a key step in PCNL surgery, occasionally the puncturing needle may penetrate the renal parenchyma and cause bleeding, and may even lead to the injury of adjacent organs. A lack of perirenal or pararenal adipose tissue may result in the colon lying lateral to the kidney, or even behind it [23]. Previous studies reported that the occurrence rates of retrorenal colon ranged from 6.8 to 10% in the prone patients while 1.9–2% in the supine patients [24, 25]. Although there are currently no statistical reports of retrorenal colon in lateral decubitus patients, this position may contribute to the forward movement of the colon due to the effect of gravity, which could effectively avoid or reduce damage to the colon. Ultrasound-guided access allows real-time detection of the anatomical relationship of the kidney and adjacent organs, whereas fluoroscopic guidance alone may lead to inadvertent adjacent visceral organ injury and increase the risk of parenchymal and intrarenal vascular injury [26]. However, in our review, only one adjacent organ damage occurred in the prone group. Therefore, we could consider both surgical positions to be safe for PCNL procedures.

We performed this systematic review and meta-analysis to evaluate the efficacy and safety of the flank position versus the prone position during the PCNL procedure. Yet, some limitations exist in our study. First, only four RCTs were available for quantitative analysis, and the rest of included study was low-quality prospective cohort studies. And more high-quality studies were needed to generate persuasive evidence. Second, the heterogeneity among the studies was significant for several parameters, which might result from the differences in sample size, flank position, detecting tools of puncture, surgical skills, outcome definitions and standards, or follow-up time among studies. Third, there was no long-term follow-up data to make our conclusion more persuasive. Perhaps a longer follow-up would reveal more significant differences between these two surgical positions. Finally, the tubeless PCNL technique was conducted in only one research [9], and there was not enough data to execute a sufficient subgroup analysis.

## Conclusions

According to our recurrent limited meta-analysis, the performance of PCNL in the flank position and prone position presented similar efficacy and safety with possibly less hemoglobin decline in the flank group. Especially, the modified lateral position could allow the extraction of complex upper urinary tract stones through simultaneous ante- and retrograde renal or ureteral access. In addition, more high-quality and well-designed RCTs with long-term follow-up are needed to increase the persuasiveness of our conclusion. Presently, majority of urologists perform PCNL in the prone position. However, the flank position is practical for PCNL, especially in patients who were obese, kyphotic, or high risks under general anesthesia. After all, the optimal surgical position should be chosen based on the patients’ conditions and urologists’ acquaintance to achieve extreme stone clearance with a safe position.

## Data Availability

Data is provided within the manuscript or supplementary information files.

## Abbreviations

PCNL: percutaneous nephrolithotomy
PRISMA: Preferred Reporting Items for Systematic Reviews and Meta-Analyses
MeSH: Medical Subject Headings
RCTs: randomized controlled trials
SFR: stone-free rate
CI: confidence interval
LE: levels of evidence
RR: risk ratio
MD: mean difference
